# Crop Mapping Using the Historical Crop Data Layer and Deep Neural Networks: A Case Study in Jilin Province, China

**DOI:** 10.3390/s22155853

**Published:** 2022-08-05

**Authors:** Deyang Jiang, Shengbo Chen, Juliana Useya, Lisai Cao, Tianqi Lu

**Affiliations:** 1 College of Geo-Exploration Science and Technology, Jilin University, Changchun 130026, China; 2 Department of Geomatics Engineering, University of Zimbabwe, Harare P.O. Box MP167, Zimbabwe; 3 Key Laboratory of Marine Mineral Resources of Ministry of Natural Resources, Guangzhou Marine Geological Survey, Guangzhou 510075, China

**Keywords:** crop mapping, CDL, DNN, ensemble learning, Google Earth Engine, Sentinel-2

## Abstract

Machine learning combined with satellite image time series can quickly, and reliably be implemented to map crop distribution and growth monitoring necessary for food security. However, obtaining a large number of field survey samples for classifier training is often time-consuming and costly, which results in the very slow production of crop distribution maps. To overcome this challenge, we propose an ensemble learning approach from the existing historical crop data layer (CDL) to automatically create multitudes of samples according to the rules of spatiotemporal sample selection. Sentinel-2 monthly composite images from 2017 to 2019 for crop distribution mapping in Jilin Province were mosaicked and classified. Classification accuracies of four machine learning algorithms for a single-month and multi-month time series were compared. The results show that deep neural network (DNN) performed the best, followed by random forest (RF), then decision tree (DT), and support vector machine (SVM) the least. Compared with other months, July and August have higher classification accuracy, and the kappa coefficients of 0.78 and 0.79, respectively. Compared with a single phase, the kappa coefficient gradually increases with the growth of the time series, reaching 0.94 in August at the earliest, and then the increase is not obvious, and the highest in the whole growth cycle is 0.95. During the mapping process, time series of different lengths produced different classification results. Wetland types were misclassified as rice. In such cases, authors combined time series of two lengths to correct the misclassified rice types. By comparing with existing products and field points, rice has the highest consistency, followed by corn, whereas soybeans have the least consistency. This shows that the generated sample data set and trained model in this research can meet the crop mapping accuracy and simultaneously reduce the cost of field surveys. For further research, more years and types of crops should be considered for mapping and validation.

## 1. Introduction

Obtaining accurate crop planting distribution information can provide important support for food security, government agricultural management, and loss assessment [1,2]. Combining remote sensing imagery and ground survey data for large-scale crop mapping proved to be an effective method as early as the 1970s and 1980s [3,4,5]. Remote sensing images have the advantages of low cost and wide coverage over ground surveys [6,7]. This technology can provide real-time, accurate, and useful information for crop surveys [4].

In recent years, various satellite image time-series data are freely accessible by the public, these include Landsat, Sentinel, MODIS, etc. These images can provide effective data support for crop distribution data products [3,4,7,8,9,10,11]. For visible and near-infrared bands, different crops have different reflection and absorption characteristics on the spectrum, which can provide rich information for distinguishing the different crop types [8,12]. Making full use of the spectral-temporal characteristics of different phenology periods for crop classification can significantly improve classification accuracy [5,13,14,15]. Many government departments and research institutions use satellite imagery and machine learning (ML) algorithms, such as support vector machine (SVM) [16], decision tree (DT) [8,17,18], random forest (RF) [3,9], and deep neural network (DNN) [17,18,19,20], for land cover and land use classification and crop mapping [18,21]. Compared with other classification algorithms, DNN has the advantage of not requiring much engineering and professional knowledge [22]. Through these methods, the United States, Canada, Germany, and China have developed crop distribution mapping products, and the accuracy of the main crops exceeds 80% [3,4,8,9,13]. Traditional multi-temporal mapping often relies on multiple time phases in a certain area, and large-scale mapping often involves complex data preprocessing processes [10]. The cloud computing platforms such as Google Earth Engine (GEE) and Amazon Web Services (AWS) provide powerful computing power for large-scale data processing [23], and can be used for crop information extraction [9,24,25]. 

Although there are many crop classification products, it usually takes several months or longer for these products to be obtained by the public. For example, USDA CDL products will not be released until February of the following year. Available crop distribution products for Northeast China are only from 2016 to 2019, and the time series is relatively short [9]. If one needs to obtain information pertaining to crop distribution for various applications such as growth monitoring or agricultural disaster losses, the process is cumbersome, costly, and tedious [26]. Technically, no matter which supervised classification method is adopted, to obtain higher classification accuracy, it is necessary to collect and process massive data samples to meet the reliability of model training and statistical analysis [10,18,24,26,27]. Traditionally, most crop distribution mapping relies on samples obtained by ground surveys, but for some regions or developing countries, large-scale mapping is a time-consuming and laborious task [15,25,28,29]. For example, the existing crop distribution products in Northeast China have only three crop types: corn, rice, and soybeans [9]. To map other crop types, it is necessary to collect own samples. Lack of training data becomes a major obstacle for various machine learning algorithms [29,30,31]. 

To address these challenges, training samples can be generated from existing historical crop distribution products and applied to other regions, to save the human and financial costs of sample collection as much as possible. Classification errors are inevitable in samples collected from historical CDL products. Obtaining samples with higher classification accuracy from these collected samples is the premise of training various machine learning algorithms. This paper proposes a DNN-based ensemble learning (EL) method to generate the required samples for these algorithms. Ensemble learning completes the classification task by combining multiple classifiers, which have higher accuracy than a single classifier [32,33]. The final results of multiple classifiers are generated by the majority voting method [34]. The objective of this study is to systematically generate hundreds of thousands of samples from the historical CDL data set based on ensemble learning methods for reference purposes, and then utilize them in mapping the crop distribution in the target area. We also compare and evaluate the effect of crop distribution mapping by comparing existing products and field verification points [9]. Specifically, this paper solves the following problems:

(1) Which algorithm has the highest classification accuracy for crop mapping, thus comparing four classification algorithms namely SVM, DT, RF, and DNN?
(2) Which month of the crop’s entire growing period has the highest crop mapping accuracy?
(3) What is the accuracy of crop mapping during the entire growing period of the crop?
(4) How consistent is the generated product compared to the existing product?

## 2. Materials and Methods

### 2.1. Study Area

**Figure 1:**
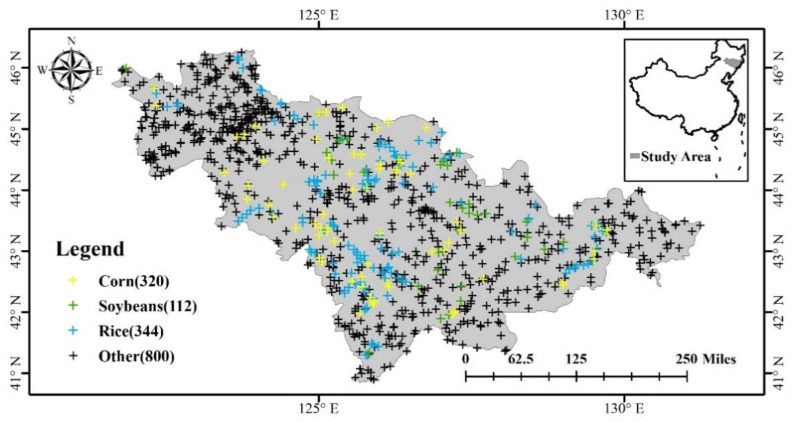
Study area and validation point distribution. Numbers in parentheses indicate the number of sample points.

The study area is located in Jilin Province and is in Northeast China (Figure 1). It is an important grain production base in China [9,35]. It is bounded between latitudes (40°52′~46°18′ N) and longitudes (121°38′~131°19′ E). It can be divided into three major landforms: the eastern mountain and the central and western plains. Jilin Province belongs to a temperate continental monsoon climate with four distinct seasons. The annual precipitation is 400–900 mm, the average temperature is 2–6 °C, and the soils are very fertile making them suitable for crop growth [36,37]. The main food crops in Jilin Province are corn, rice, and soybeans. 

### 2.2. Material

#### 2.2.1. Remote Sensing Data

Sentinel-2 (European Space Agency) time series images corresponding to the crop growing season from May to October are the main data source for crop mapping. Sentinel-2 consists of two satellites, Sentinel-2A and Sentinel-2B with a high spatial resolution and short revisit period of 5–6 days, hence more opportunities to obtain time-series images. It carries multi Spectral Instrument (MSI) onboard that contains 13 spectral bands ranging from visible and near-infra-red (VNIR) bands and short wave infra-red (SWIR) bands [38]. 

Remote sensing image preprocessing and data acquisition are conducted mainly through the Google Earth Engine (GEE, Google, Menlo Park, CA, USA) platform. GEE provides a wealth platform for open data for research on a wide range of land cover classification and crop extraction [39]. Sentinel-2 (S2) atmospheric top reflectivity products are utilized and images with cloud cover of less than 50% were selected, masked with quality control bands, and composited by median [39]. There, however, are still some missing pixels in some areas of the month. For a missing pixel, the average value of the two phases before and after is used for time series interpolation. Each pixel is then filtered using Savitzky–Golay algorithm to reduce the effect of noise [40,41]. To ensure that the resolution and projection of the image and the corresponding CDL data are consistent, the data are resampled to 30 m, and the WGS84 coordinate system is adopted.

#### 2.2.2. Ground Truth Data

The 30 m resolution crop data layer (CDL) data sets corresponding to remote sensing images were obtained from the United States Department of Agriculture (USDA) National Agricultural Statistics Service (https://www.nass.usda.gov/Research_and_Science/Cropland/Release/, accessed on 1 July 2022). The data provide acreage estimates according to the NASS Agricultural Statistics Board (ASB) and Field Offices (FOs). These data sets are created from medium-resolution satellite images, such as Landsat, combined with ground survey data and machine learning algorithms such as decision tree algorithm training data. The data sets include more than 110 crop types. The satellite data is used to monitor the entire phenological phase of crops. The classification accuracy of main crop types is between 85% and95%, whereas the classification accuracy of corn exceeds 95% [4].

#### 2.2.3. Validation Data

Two types of data are used to evaluate the results of the extracted crop mapping. The first data set is the crop map of Northeast China published by You et al. [9] from 2017 to 2019. The data set was generated using a random forest classification algorithm for crop types including corn, rice, and soybeans, with overall accuracies (OA) ranging from 0.81 to 0.86 and at a spatial resolution of 10 m. It was resampled to 30 m resolution according to the median method. For ease of description, authors are calling this data Northeast China, and the crop classification product generated in this paper is called EL-DNN. The second data set was collected by GPS equipment in 2017 and 2018 for corn, soybeans, and rice as shown in Figure 1. A total of 800 sample points were marked from Google Earth as a compliment. The GPS samples were used for accuracy assessment in addition to Northeast China data set.

### 2.3. Methodology

**Figure 2:**
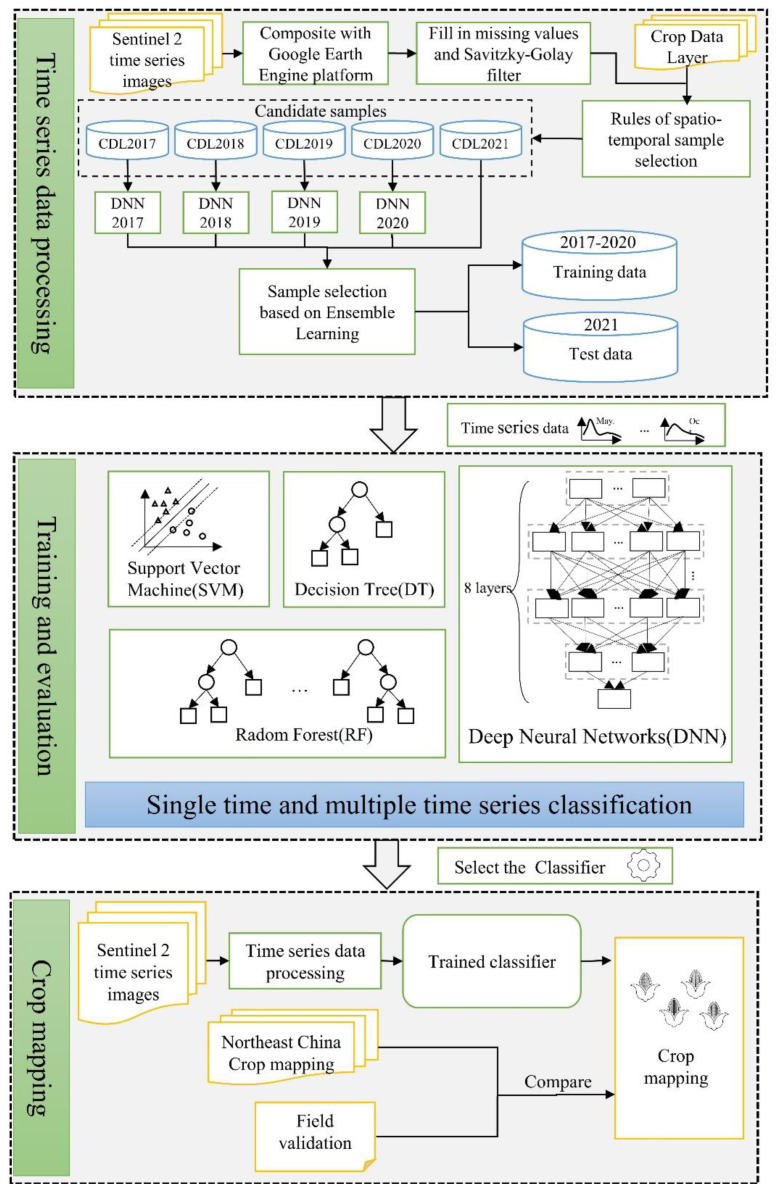
Flowchart for the crop mapping.

The flowchart (Figure 2) presents processes followed in the crop distribution mapping and is in three main steps. Firstly, preprocessing of Sentinel-2 time-series data, and combining it with CDL data to generate a training data set and test set. Secondly, training various machine learning algorithms and comparing and evaluating the accuracy. Finally, the model with the highest accuracy was selected for crop distribution mapping, and compared with existing products to verify the consistency.

#### 2.3.1. Time Series Data Processing

For the preprocessing of time-series, sufficient samples were obtained based on the spatiotemporal sample rules for the classifiers The spatiotemporal sample selection method is based on two rules: (i) Due to crop rotations on planting area, same areas in even and odd years as sample candidate areas, respectively, were maintained. (ii) The features corresponding to the pixels in the larger fields have higher accuracy. For these two rules to be applicable, there is a need to ensure that the collected samples have a high degree of confidence. Sample points were randomly generated, and a continuous area with the same crop type of 5 × 5 pixels was selected, and the points falling in this area were selected as the final crop type sample point. Since the recognition of pixels is usually affected by the proximity effect, this method avoids the impact of mixed pixels on the classification of different terrain boundaries [4,7]. The procedure was repeated many times until a certain number of cycles was met or a certain number of sample points is reached. Using GEE, samples were generated by extracting the values corresponding to each point from Sentinel-2 and CDL data. Since the main crop types in the study area are corn, rice, and soybeans, authors reclassified the crop type numbers corresponding to the CDL (Table 1). 

Five-year candidate sample data are generated from historical CDL data according to the spatiotemporal sample selection method, and about one million samples were collected each year. The samples from 2017 to 2020 were used as the training candidate sample set and the samples in 2021 were used as the test data candidate sample (CDL2021). Since the samples are derived from historical CDL products, there are inevitable misclassified samples. This paper proposes a method of using ensemble learning to generate high-precision sample sets. Ensemble learning combines multiple classifiers to obtain classification results with high accuracy [34,36,37]. Therefore, with this method, the samples with low classification accuracy are removed from the candidate samples, and the remaining samples with high classification accuracy are reserved for training data sets. 

Considering a candidate sample in 2017 as an example. Firstly, the candidate samples from 2017 to 2020 are used to train four DNNs models; then, select the DNNs trained in 2018, 2019, and 2020 to evaluate the category of this candidate sample. If the three results are consistent, the sample is retained, otherwise, it is discarded; similarly, for a candidate sample in 2018, the models of 2017, 2019, and 2020 are selected for candidate sample determination. This process is repeated until the four-year samples are judged, and the training data set is finally generated. For the candidate sample set (CDL2021) generated in 2021, use the four DNNs previously trained to determine each candidate sample to obtain the final test data set (EL2021). 

#### 2.3.2. Training and Accuracy Evaluation

To compare the performance of the different machine learning algorithms, the accuracy assessment was conducted on the test sets of four widely used land cover/use and crop classification algorithms, namely SVM, DT, RF, and DNN [3,8,9,16,17,18,19,20,42,43]. SVM, DT, and RF use the algorithm provided by python’s scikit-learn. DNN mainly relies on the Keras and Tensorflow frameworks and is trained on the GPU, which can have a faster calculation speed than the CPU. A multi-layer deep neural network model with the number of neurons in order of 16, 16, 32, 32, 64,32, 32,10, batch normalization is performed before each layer is input, and a dropout layer is added after each layer to prevent model training from overfitting [44,45,46]. The final selection of the highest probability value is considered the classification result. For a single month, the spectral characteristics of the corresponding month are entered, and for multiple months, the time series is used as the input. The core of network parameter optimization uses a backward propagation algorithm [47,48]. In this paper, the adam algorithm [49] is used to train and optimize the network.

Classification accuracy evaluation indicators considered are the kappa coefficient, overall accuracy (OA), and confusion matrix [24,50]. The former two are used as the overall accuracy index of the classifier for each category, and the confusion matrix is used to analyze the misclassification of corn, soybeans, rice, and other coverage types. Each column in the confusion matrix represents the predicted class, each row represents the true attribution class of the data, and all correct predictions are located on the diagonal of the table. The calculation of kappa coefficient is based on the confusion matrix, and the calculation formula is:

(1) $$kappa=\frac{N\sum_{i=1}^{r}x_{ii}-\sum_{i=1}^{r}\left(x_{i+}\times x_{+i}\right)}{N^{2}-\sum_{i=1}^{r}\left(x_{i+}\times x_{+i}\right)}\;,$$

where r is the number of rows of the confusion matrix; $x_{ii}$ is the number of type combinations along the diagonal; $x_{i+}$ is the total number of observations in row i; $x_{+i}$ is the total number of observations in column i; N is the total quantity.

In order to better visualize the misclassification of each category, it is expressed as a normalized confusion matrix [24]. Each row of the matrix represents the percentage of the true value that is divided into other categories. The values on the diagonal of the matrix are the producer accuracy (PA), which indicates the percentage of the correct classification of the category to the total. The last row of the normalized confusion matrix represents user accuracy (UA), which is the proportion of the correct number of classifications in the classifier. Overall accuracy (OA) represents the proportion of all correctly classified samples to the total number of samples.

#### 2.3.3. Crop Mapping

The Sentinel-2 images were preprocessed and the trained classifier was used for crop distribution mapping in the study area and compared with Northeast China [9] crop distribution products. In the process of mapping, we found that some types of wetlands would be mistakenly classified as rice. In response to this situation, we proposed a correction method combining two time-series data of different lengths. The accuracy and consistency of the two products were compared by collecting and verifying sample points. 

## 3. Results

### 3.1. Generating Sample Data Sets Based on Ensemble Learning

Select the classifier with the highest accuracy from SVM, DT, RF, and DNN as the basic classifier for ensemble learning. Various hyperparameters are optimized using sklearn’s GridSearch CV. GridSearch CV adopts five-fold cross-validation and selects the hyperparameters with the highest accuracy as model parameter values, and the rest of the parameters as the default values. Table 2 shows the hyperparameters optimized by the four machine learning algorithms throughout the growth period on the candidate sample data set. When the model inputs data of other time lengths, the same GridSearch CV method is used for each parameter selection of SVM, DT, and RF. Each model parameter of DNN selects the fixed value in the table. This is because DNN requires longer training time than other models, and it consumes a lot of computing resources and time to perform parameter tuning of each model. This paper generates a large number of samples from historical data for tuning various parameters of DNN, even if different hyperparameter combinations will eventually converge. This may be due to the fact that DNN can express more complex functional relationships than other machine learning algorithms, and different parameter combinations can achieve the same effect. It can be seen from the table that DNN has the highest accuracy compared with other algorithms kappa and OA.

In order to obtain samples with higher classification accuracy for the training of machine learning algorithms such as deep neural networks, the candidate sample data set (CDL) is generated from the historical data using the spatiotemporal sample selection method, and the data set (EL) with high classification accuracy is obtained through ensemble learning. Table 3 shows the average removal percentage of the four classification algorithms in different years. DT and SVM have more samples removed, accounting for 47% and 37% on average in four years. This is related to poor classifier performance, and different classifiers have more inconsistencies, resulting in a large number of samples being removed. The average percentage of DNN and RF removals is basically the same, with RF only 1% higher. More samples were removed in 2017 and 2021 than in other years. This may be related to the lower overall accuracy of non-agricultural types in 2017. In 2021 it is voted by four classifiers, and a relatively large number of inconsistent samples will be cast. Table 4 shows the relative percentages of each category removed from the candidate sample by DNN-based ensemble learning. The overall average removal rate of each category in five years is 20%. The three main crop types had fewer sample removals than the other land cover types. Among the three crops, soybeans removal was the most at 17%, and rice removal was the least each year, with an average of 6%. Wetlands had a high number of removals each year, with a five-year average of 40%, and about 30% of the remaining types of samples were removed. This shows that when the candidate samples of each year are used for model training, the remaining land cover types have more uncertainties in the classification than the three crop types.

**Figure 3:**
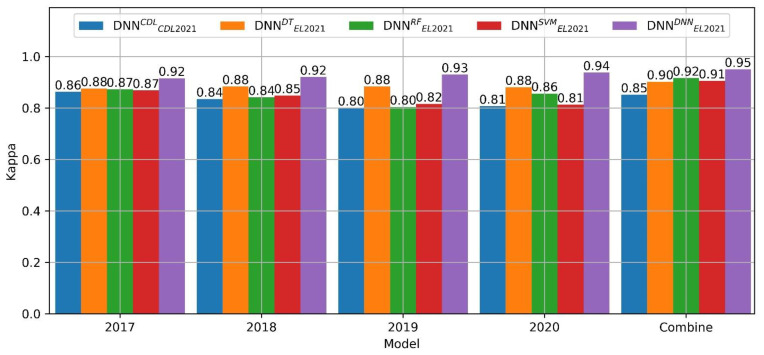
Kappa coefficients for models trained on candidate and ensemble learning samples. Each year represents the model trained on the samples of the corresponding year. The superscripts denote the candidate sample data set (CDL) and the sample set generated by ensemble learning with four classification algorithms. The subscripts CDL2021 and EL2021 denote the candidate test set and the ensemble learning generated test set, respectively. Combine means combining four years of data for training.

Figure 3 shows the comparison of training DNN accuracy on data sets generated by ensemble learning of different classifiers. Overall, the models trained by the samples obtained through ensemble learning have a higher kappa coefficient each year than the models trained by the candidate samples. After combining four years of training data, the accuracy of the models trained by the four algorithms is improved compared to the accuracy of a single year. Among them, the sample kappa coefficient generated by DNN-based ensemble learning is up to 0.95, and the other three algorithms are all higher than 0.9. It is about 0.1 higher than the candidate sample test set. This shows that the training data set with higher accuracy can be obtained through the ensemble learning method. Therefore, this paper selects the sample set generated based on DNN ensemble learning for different model classification performance evaluations and final crop distribution mapping model training.

### 3.2. Comparison of the Accuracy of Different Classifiers

**Figure 4:**
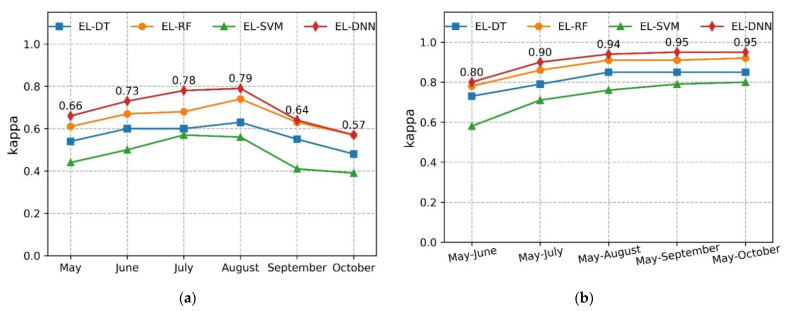
Kappa coefficient of different classification algorithms on the test data set (EL2021). (**a**) Single month kappa coefficient; (**b**) monthly time-series kappa coefficient.

The generated training data set samples were used to train four classification algorithms namely EL-DT, EL-RF, EL-SVM, and EL-DNN, and the kappa coefficient is used as the overall accuracy classification index on the test data set to evaluate the performance of the four algorithms. Compared with EL-DT, EL-RF, and EL-SVM, EL-DNN has the highest accuracy in both single months and multiple months (Figure 4). For a single month, the four algorithms had higher classification accuracy in July and August compared to the other months. EL-DNN has high classification accuracy in July and August with kappa coefficients of 0.78 and 0.79, respectively (Figure 4a). This indicates that this period is the best for discriminating against various land cover types and crop types. 

For monthly time series, the classification accuracies for the four algorithms gradually increase as the length of the time series increases, which is significantly higher than a single month (Figure 4b). The kappa coefficient of the entire crop growth period reached the highest value of 0.95. After August, there is a subtle increase in the accuracy, thus, only 0.01. Therefore, better crop distribution maps to obtain can be obtained before August.

**Figure 5:**
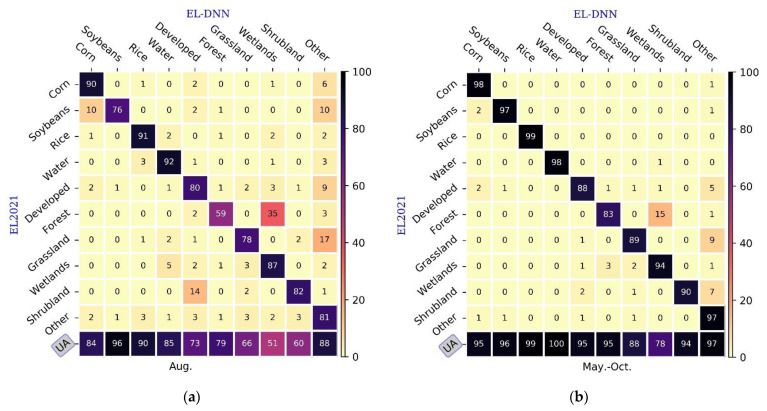
Normalized confusion matrix on the test data set (EL2021). (**a**) August; (**b**) May to October.

EL2021 data were used for the ground-truthing, and the normalized confusion matrix to analyze the classification of corn, soybean and rice, and other land cover types (Figure 5). Figure 5a shows the normalized confusion matrix for the classification of various land types in August. At this stage, rice had the best classification accuracy among the three crop types, with PA of 91% and UA of 90%. Although corn PA reaches 90%, about 6% of corn was classified as other types. Meanwhile, about 10% of soybeans and 2% of developed and other types of samples, respectively, were classified as corn, resulting in a lower UA of 84%. This is because different crops may have a similar phenological period to corn, making it difficult to distinguish. Soybeans had the lowest classification accuracy, with 10% being classified into corn and other types, respectively. Overall, for single-month crop classification, rice had the highest classification accuracy among the three crop types, followed by corn and soybeans with the least. 

There were misclassifications among other land cover types. Compared with August, the classification accuracies of the three main crops and other land cover types improved during the whole growing period from May to October (Figure 5b). The PA and UA of the three crop types were all higher than 95% and were well discriminated. About 1% of the samples of other types, corn and soybeans were misclassified from each other. The misclassification of other land cover types was significantly lower than that of a single month. This shows that time series can significantly improve the classification accuracy of various categories and reduce the number of misclassified categories. Therefore, making full use of the phenological characteristics of different vegetation types can improve the accuracy of crop classification. 

### 3.3. Comparison between EL-DNN and Northeast China Products

**Figure 6:**
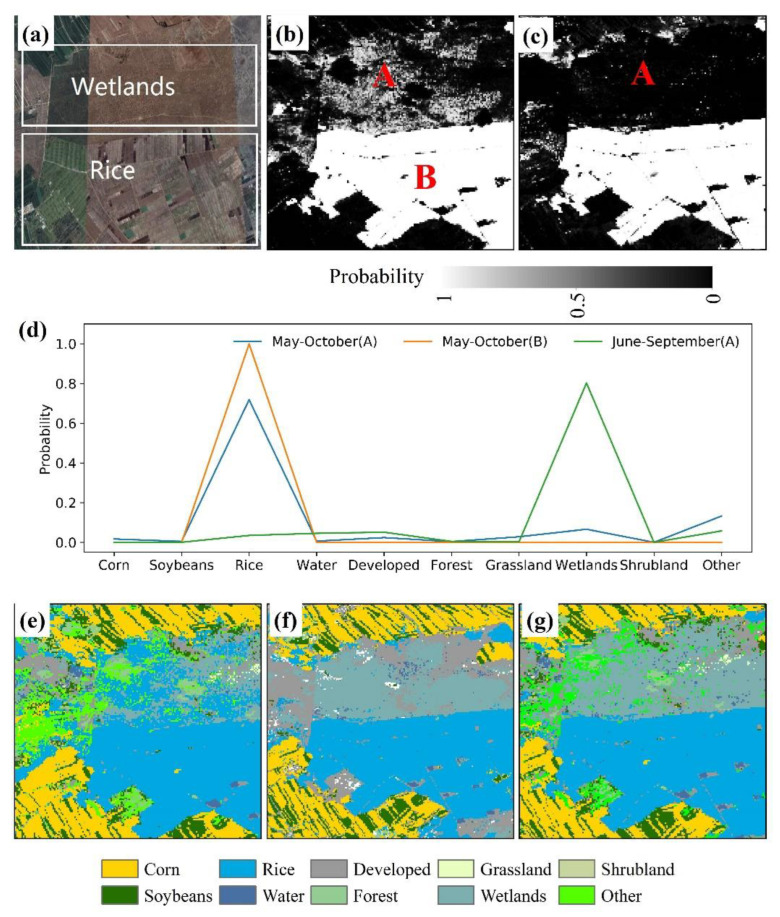
Correction of rice classification combined with phenological sequence sequences of different lengths. (**a**) Google Earth image on 4 September 2019, including wetlands and rice; (**b**) May–October rice category probability values throughout the growth period, where A and B are the two locations representing wetlands and rice, respectively; (**c**) June–September. Rice category probability; (**d**) comparison of the probability values of each category in the two positions A and B; (**e**) May-October classification results of the whole growing period; (**f**) June–September classification results; (**g**) merge the length-corrected results of the two-time series.

During the classification and mapping process, wetland areas were misclassified as rice (Figure 6). This may be due to the early precipitation of the wetland, which has similar spectral characteristics to rice, and the model is less discriminative. For example, Figure 6a is a Google Earth high-resolution map, from visual inspection, it can be seen that the rice and wetland areas in this area have distinct spatial characteristics. Rice in the wetland area during the whole growing period from May to October had a higher probability value (Figure 6b). The probability values decreased in the wetland area from June to September (Figure 6c). For example, select two points A and B to compare the probability values of each category output by the model (Figure 6d). A is a wetland type, and the probability value of each category in the whole growth period is higher but is slightly lower than that of rice B. When June to September period is selected, the probability value of wetland is higher and the rice type is lower. Figure 6e,f shows the classification results of time series of different lengths. Therefore, the entire sequence of rice can be corrected using the data from June to September. Figure 6g shows the corrected rice classification results, so it can be seen that the misclassified areas of wetlands are corrected.

Corn, rice, and soybean sample points collected in 2017 and 2018 were used to assess the consistency of our generated crop distributions in Northeast China (Table 5). Overall, the OA and kappa coefficients of the two-year EL-DNN are slightly higher than those of Northeast China’s products. In 2017 and 2018, the Kappa coefficient of EL-DNN was 0.06 and 0.03 higher than that of Northeast China products, and the OA was 3% and 1% higher, respectively. Except for the other type, the PA and UA rice were higher among the three crop types in the two years. The second was corn, and the soybean was the worst. In conclusion, the generated products are generally in good agreement with the accuracy of the northeast region.

**Figure 7:**
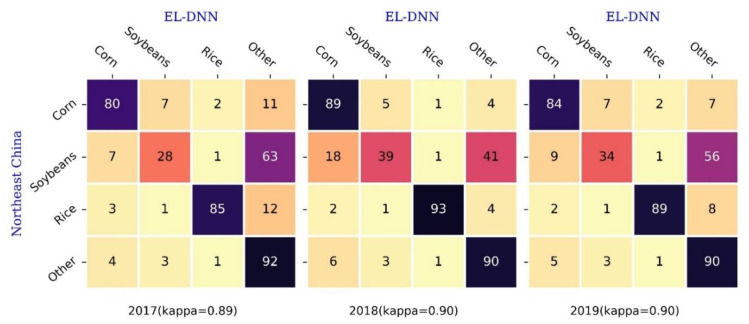
Normalized confusion matrix assuming Northeast China is true from 2017 to 2019.

Figure 7 compares the consistency of EL-DNN and Northeast China crop distribution products in 2017–2019. Since EL-DNN has more categories than Northeast China, for the sake of convenience, the remaining categories except corn, soybean, and rice are merged into the other category (EL-DNN Merge). Then, with the crop distribution data in the northeast as the ground truth, various types of differences were overall assessed in the form of a normalized confusion matrix. The average value of various comprehensive kappa coefficients within three years is 0.9. Among the three crop types, the annual PA of rice is above 85%, followed by corn, all above 80%, and soybean is the worst, the highest being only 34%. There are many inconsistent values between northeast soybean and EL-DNN other types. To sum up, with the exception of the other type, the two products had the highest consistency in rice, followed by corn, and the worst in soybean.

**Figure 8:**
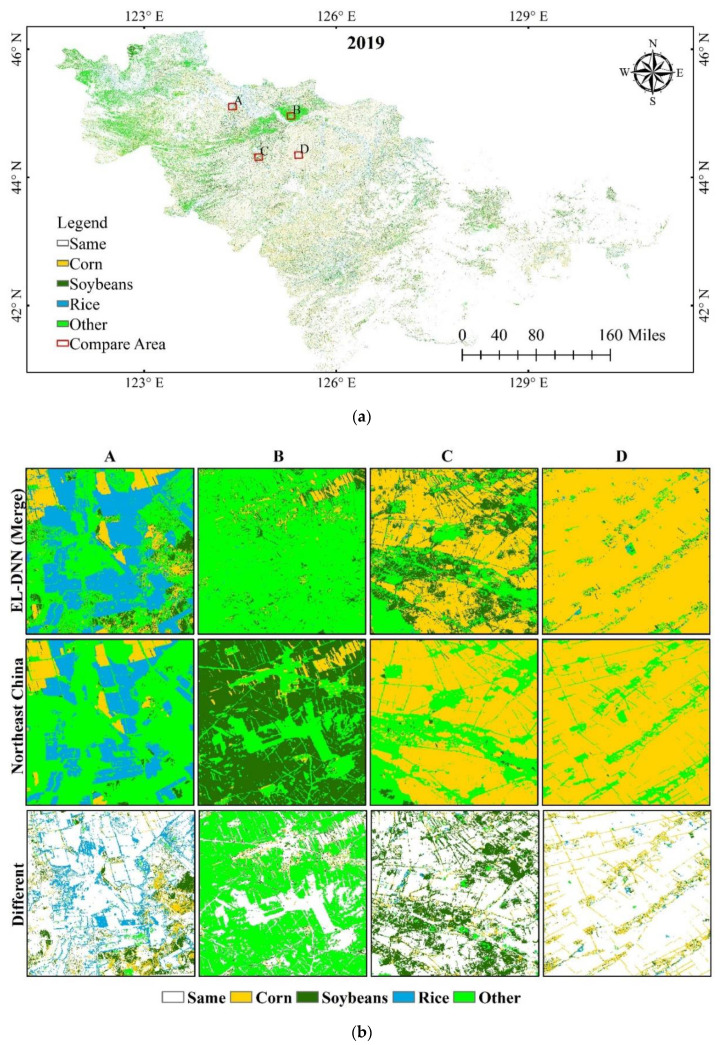
Different pixels of EL-DNN and northeast products. (**a**) Different pixels of the two products in 2019; (**b**) four regions of A, B, C, D in 2019.

Using the generated samples and the trained DNN model, we generated crop distribution products at 30 m resolution in Jilin Province from 2017–2021. The data set can be obtained through https://github.com/Jiang2019Code/JilinCropMapping, accessed on 1 July 2022. Figure 8 shows the difference in pixel spatial distribution between EL-DNN crop mapping and Northeast China in 2019, and selects the four regions of ABCD to compare the differences between the two products. It can be seen from the figure that most of the different areas are located in the western part of the study area, and the remaining areas have a relatively good consistency (Figure 8a). Although we corrected for rice, the two different areas in area A are rice (Figure 8b). There are larger differences at the edges, possibly due to mixed pixels. Other types and soybeans are misclassified with each other, such as regions B and C. As in Figure 7, there are large differences between the two soybean products. Corn in region D has high consistency, but there is inconsistency in the edge region. This may be because the Northeast China product data are 10 m, the DNN is 30 m, and the mixed pixels lead to misclassification of the results.

## 4. Discussion

The authors used historical CDL data from 2017 to 2020 to generate sample data sets to train and evaluate DNN for crop distribution mapping in the study area. The sample data set plays a vital role in the entire classification [28,51], but sample data set acquisition can be an expensive task [13,27,52]. A variety of representative samples can make the classifier have better discrimination capabilities which influence the final classification accuracy [53,54]. Ideally, if the physiology and growth environment of crops are the same in all image acquisition phases in different regions, then crops should have similar spectral characteristics. However, because it is based on composite data at a certain time interval and the influence of internal and external environments, crop phenological characteristics change with location and seasons [52]. Therefore, the use of machine learning algorithms for large-scale mapping is inseparable from the support of massive sample data.

Samples collected from historical CDL data are inevitably misclassified, and the internal uncertainty of the data set will bias the classification results [10,55]. Compared with the method of collecting samples manually, the method based on spatiotemporal sample selection rules generates a large number of candidate representative samples based on the historical CDL data set [4,10]. The samples whose classification accuracy is obtained from the candidate samples by means of ensemble learning constitute the training data set (Figure 3). In addition, when long-term monitoring of crop cultivation changes is required, earlier satellite data support is required [9]. On the one hand, we have been unable to collect samples on the ground. On the other hand, compared with Sentinel-2, the Landsat series satellites have a longer revisit period and cannot obtain continuous time-series images. Remote sensing images usually select the most sensitive time phase for crop phenology, which can effectively distinguish different crop types [2,20,52]. At this point, we can select images from July and August first, and use the sample data set we generated to select the corresponding band to train the model for crop distribution mapping.

The constructed DNN achieve higher accuracy than other machine algorithms on the test data set (Figure 4). This may be related to the fact that we have collected a large number of samples. For traditional machine learning algorithms, when the number of samples reaches a certain number, the accuracy no longer increases. The performance of DNN continues to increase with the increase in sample size until the maximum value [56,57]. Multi-temporal images with different phenological periods can achieve higher accuracy than a single phase [13,14,26]. Multiple monthly time series can provide the model with more spectral features and improve classification accuracy (Figure 4 and Figure 5). As the time series grows, we find that the classification accuracy is effectively improved. In general, the kappa coefficient of various types of features was as high as 0.94 in August compared with a single phase, and the increase was not obvious after that. On the test set, the kappa coefficient is higher than the existing research 0.86 [26]. For corn, soybeans, and rice, more than 95 percent accuracy can be achieved as early as August. This period can be used as a key period for crop classification, which is close to the existing research [13].

The results generated were in good agreement with Northeast China products for rice and corn, and relatively poor for soybeans (Figure 7 and Figure 8). Through the verification of the collected samples (Table 5), the accuracy was close to that of Northeast China products [9]. However, compared to the test data set, the accuracy of the three crop types on the validation set are relatively low. This may be due to differences in regions, soil background environments, and phenological periods, resulting in spectral differences that reduce the generalizability of the model. On the validation set, the classification accuracy of soybean products generated in this paper is better than that of Northeast China, but still lower than that of corn and rice. This is because soybeans are easily confused with other types, such as peanuts, sunflowers, etc. [9]. In the western part of the study area, there are great differences between the two products, and more samples need to be collected for comparison in this area (Figure 8a). During the classification process, precipitation easily misclassifies wetlands as rice. This can be corrected by combining two time series of different lengths (Figure 6). In addition, there were misclassifications among other surface types due to changes in surface cover types throughout the growing period. For example, for bare land, the surface vegetation will change and the other type will be confused. Overall, the non-agricultural land cover is less accurate than the crop type because the non-agricultural land cover in CDL relies on the USGS National Land Cover Data Set (NLCD). The overall accuracy is the highest at 89% [58]. This can also indicate that ensemble learning has more samples removed (Table 4).

Misclassifications are prone to occur for bounded regions because some regional plots in the northeastern region are scattered and relatively small. The boundary pixels of the plots contain a variety of features, and mixed pixels will further affect the classification effect [21], such as Figure 8b. The time series of a single-pixel only considers the information of a single pixel, while ignoring the spatial features between adjacent pixels. The same crop has internal homogeneity, and boundary heterogeneity can effectively distinguish crop boundaries. In the future, it is possible to improve the classification accuracy of the boundary by increasing the texture features of the image [9]. We only collected three crop types of corn, soybean, and rice for accuracy evaluation, the remaining types still have good classification accuracy on the test set, such as woodland, water body, wetland, etc. (Figure 5), we will do more in the future crop mapping and sample collection were used for validation.

## 5. Conclusions

In the context of global population growth and increasing natural disasters, mastering the distribution of major food crops provides useful information for taking active countermeasures. In the field of remote sensing image classification, the training of various algorithms requires massive sample training sets (such as DNN) and there is a contradiction in the high cost of obtaining sample training sets. To solve this problem, this paper proposed two strategies to obtain massive samples with high classification accuracy. First, the spatiotemporal sample selection rules can generate a large number of candidate samples from the historical crop data layer. Then use ensemble learning to obtain training samples with higher classification accuracy from candidate samples. Based on the generated training sample set, the classification accuracy of different algorithms is evaluated. The main conclusions are as follows:

1. The error of data labeling is the main factor affecting classification accuracy. The kappa coefficient of the classification accuracy of the samples selected by ensemble learning in the whole growth period is improved from 0.85 to 0.95. Most of the removed samples are non-agricultural land covers with large errors. This shows that the ensemble learning method can obtain high-precision samples and remove most of the mislabeled samples.
2. Compared with the four main machine learning algorithms, DNN is higher than DT, RF, and SVM. This proves that DNN can learn more complex features and has significant advantages in processing massive data.
3. Comparing the results of single-phase and multi-phase classification, July and August have the highest accuracy, which can be used as the key period for crop classification. During the whole growing period of crops, with the growth of the time series, the classification accuracy of the three crop types was higher than 95% until August, and with the growth of the time series, the overall accuracy did not improve significantly.
4. The crop distribution products generated by our method can achieve relatively consistent accuracy with the existing products. Rice and corn have higher consistency, and soybean consistency is poor. Wetlands may be misclassified as rice due to the influence of precipitation, which can be corrected by combining the classification results of sequences of different lengths. Soybeans are more difficult to distinguish from other crop types.

In short, the model trained based on the generated sample data set can meet the needs of crop distribution mapping update, without spending a lot of time and manpower on type labeling. Based on the same method, this data set can be applied to other regions or crop types for mapping, which has a certain application potential.

## Figures and Tables

**Table 1:** Crop category, value, and number of samples.

CDL Value	New Value	Name
1 (Corn), 12 (Sweet Corn), 13 (Pop or Orn Corn)	1	Corn
5 (Soybeans)	2	Soybeans
3 (Rice)	3	Rice
83 (Water), 92 (Aquaculture), 111 (Open Water), 112 (Perennial Ice/Snow)	4	Water
121 (Developed/Open Space), 122 (Developed/Low Intensity), 123 (Developed/Med Intensity), 124 (Developed/High Intensity)	5	Developed
63 (Forest), 141 (Deciduous Forest), 142 (Evergreen Forest), 143 (Mixed Forest)	6	Forest
176 (Grassland/Pasture), 59 (Sod/Grass Seed)	7	Grassland
87 (Wetlands), 190 (Woody Wetlands), 195 (Herbaceous Wetlands)	8	Wetlands
64 (Shrubland), 152 (Shrubland)	9	Shrubland
Other values	10	Other

**Table 2:** The optimal hyperparameters settings and highest accuracy for SVM, DT, RF, and DNN.

Classifier	Parameters	Description	GridSearch Values	Search Result	OA	Kappa
SVM	C	Regularization parameter	0.01, 0.1, 1, 5, 10, 50, 100, 500	500	0.71	0.65
DT	Criterion	The function to measure the quality of a split.	Gini, entropy	Gini	0.81	0.76
	max_depth	The maximum depth of the tree.	10, 50, 100, 200	50		
	min_samples_leaf	The minimum number of samples required to be at a leaf node.	10, 50, 100, 200	10		
	min_impurity_split	Threshold for early stopping in tree growth.	0.001, 0.01, 0.1	0.001		
RF	n_estimators	The number of trees in the forest.	50, 100, 200	100	0.85	0.83
	max_depth	The maximum depth of the tree.	10, 50, 100, 200	100		
	min_samples_leaf	The minimum number of samples required to be at a leaf node.	10, 50, 100, 200	10		
	min_impurity_split	Threshold for early stopping in tree growth.	0.001, 0.01, 0.1	0.001		
DNN	learning_ rate	Learning rate schedule for weight updates.	0.001, 0.01, 0.1	0.001	0.88	0.85
	optimizer	Optimizer algorithm.	SGD, Adam	Adam		
	activation	Activation function of the hidden layer.	relu, tanh	Tanh		
	layers	Number of network layers.	5, 7, 8	8		
	Batch	Randomly sampled for each training.	1000, 4000, 8000	8000		

**Table 3:** The percentage of samples removed by the ensemble learning of the four algorithms each year. ($\frac{Count_{CDL}-Count_{EL}}{Count_{CDL}}\times 100\%$).

Year	SVM	DT	RF	DNN
2017	42	52	25	24
2018	36	45	19	18
2019	36	46	18	16
2020	37	45	18	17
2021	36	48	24	25
Mean	37	47	21	20

**Table 4:** The percentage of each type of sample removed by DNN-based ensemble learning. ($\frac{Count_{CDL}-Count_{EL}}{Count_{CDL}}\times 100\%)$.

Year	Corn	Soybeans	Rice	Water	Developed	Forest	Grassland	Wetlands	Shrubland	Other
2017	17	24	12	24	34	39	24	45	30	23
2018	8	17	4	14	27	42	32	52	32	15
2019	13	9	7	12	30	22	41	27	20	13
2020	9	17	6	14	27	27	34	48	32	14
2021	15	22	5	13	33	38	28	32	32	30
Mean	12	17	6	15	30	33	31	40	29	19

**Table 5:** Comparison of EL-DNN and Northeast China products for various types of PA (%), UA (%), OA (%), and Kappa coefficients.

Year	Indicators	EL-DNN				Northeast China			
		Corn	Soybeans	Rice	Other	Corn	Soybeans	Rice	Other
2017	PA	79	63	84	98	82	57	90	93
	UA	76	64	98	95	66	37	95	96
	OA	93				90			
	Kappa	0.84				0.78			
2018	PA	86	72	87	98	88	73	82	96
	UA	87	54	96	98	88	48	94	98
	OA	93				92			
	Kappa	0.87				0.84			

## Data Availability

The crop mapping data can be obtained through https://github.com/Jiang2019Code/JilinCropMapping, accessed on 1 July 2022.
